# Supplementary material for: Overcoming the Chemical Complexity Bottleneck in on-the-Fly Machine Learned Molecular Dynamics Simulations
Source: J Chem Theory Comput. 2024 Jul 8;20(14):5788–95. doi: 10.1021/acs.jctc.4c00474 (PMC11270744; doi:10.1021/acs.jctc.4c00474)
Supplement: Supplementary file 1 — ct4c00474_si_001.pdf [file ct4c00474_si_001.pdf]

Supporting Information: Overcoming the chemical complexity bottleneck in on-the-fly machine learned molecular dynamics simulations

Lucas R. Timmerman,[†] Shashikant Kumar,[‡] Phanish Suryanarayana,[‡,¶] and Andrew J. Medford[*,†]

[†]*School of Chemical & Biomolecular Engineering, Georgia Institute of Technology, Atlanta, GA 30332*

[‡]*School of Civil & Environmental Engineering, Georgia Institute of Technology, Atlanta, GA 30332*

[¶]*School of Computational Science and Engineering, Georgia Institute of Technology, Atlanta, GA 30332*

E-mail: ajm@gatech.edu

1 Supporting Information

1.1 Normalized GMP Descriptor

The GMP descriptor, originally published by Lei. et. al., is a universal mapping from Cartesian space to chemical descriptor space that embeds elemental identity via Gaussian approximations to pseudopotential valence electron densities. The original formulation of the descriptor was not suitable for on-the-fly kernel based ML as the units of the descriptors and hence magnitudes varied wildly. To overcome this issue, we apply a simple normalization factor computed at the beginning of every simulation based on the selected radial $g_n(r)$ and angular Y_m probe functions:

$$A_{n,m} = \frac{1}{\sqrt{\iiint g_n^2(r) Y_m^2(\theta, \phi) dr d\theta d\phi}}$$

where $g_n(r)$ represents a Gaussian with a specified standard deviation of n and Y_m represents the collection of Maxwell Cartesian Spherical Harmonics of order m. This scaling factor ensures uniformity in units across different angular channels and forces that magnitude of higher order descriptors to 0 as in traditional multipole expansions. This step allowed us to use traditional kernel methods to evaluate the similarity between chemical environments represented by the GMP descriptors.

1.2 Additional Computational Details

Primitive `fcc` unit cells were constructed for Ag, Al, Au, Ir, Pd, Pt, and Rh with initial guesses for lattice parameters taken from the Materials Project. The lattice constants were determined utilizing the `fmin` function from `sklearn` to minimize the energy as a function of lattice constant. Super cells were created from relaxed primitives by repeating cells. The alloys were formed by randomly replacing atoms in a Pt super cell with ∼equimolar amounts

of the alloyed elements. The mixing was done element-wise proceeding alphabetically, resulting in five random bulk alloys (PtAg, PtAgAu, PtAgAuIr, PtAgAuIrPd, PtAgAuIrPdRh). Initial guesses for the lattice parameters of the super cells were formed via composition-weighted averages of the lattice constants for the constituent elements. Relaxations on the cell and positions were carried out iteratively until both the maximum pressure in the cell fell below 1E-02 GPa and the maximum force component fell below 6E-04 Ha/Bohr. Relaxed cells were used as starting points for all MD simulations, both AIMD and on-the-fly. We used the isokinetic (NVK) ensemble with a Gaussian thermostat to maintain the simulation temperature at 500K. A time step of 2 fs was used for integration. The simulations were run until 10,000 integration steps were completed, resulting in a simulation length of 20 ps. This provided an adequate balance between a desire to minimize computational cost and the need to robustly characterize the stability of the simulations. We ran all calculations with a k-point density of 32 Å which corresponds to a k-point grid of (8,8,8) for the primitive fcc cells and (4,4,4) for the supercells. We employed mesh spacing of 0.25 Bohr which is equivalent to a plane-wave cutoff of ~1340 eV. The SCF tolerance was set to achieve at least 1E-4 Ha/Bohr accuracy in atomic forces for all relaxations and MD simulations.

We defined the TVD for integral normalized PCFs as:

$$TVD = \frac{1}{2}\int_0^{R_{max}} |g_{DFT}(r) - g_{ML}(r)|dr$$

where $g(r)$ represents the integral normalized PCF for a given interaction and the integration domain is radial distance. Normalizing the PCFs by their integral area allows us to treat them as probability density functions that describe the probability of finding a given pair of atoms at a specified radial distance from each other. This allows for a straightforward application of the TVD metric.

We define the formation energy of the alloys as the energy of the alloy minus the com-

position weighted sum of the constituent components:

$$E_f = E_{alloy} - \sum_{i}^{N_{types}} n_i E_i$$

where E_i corresponds to the per atom energy of species i in its reference state and n_i corresponds to the number of atoms of species i in the alloy. We include electronic, internal, vibrational, and configurational contributions in our calculation. The configurational entropy was computed assuming an ideal solid solution:

$$S_{conf} = -k_B \sum_{i}^{N_t ypes} x_i ln x_i$$

This term is 0 in the case of pure bulk components thereby acting as a strong driving force for the formation of the HEAs. The computation of electronic, internal, and vibrational contributions were computed differently for the FD and MD approaches. For both methods, the electronic contribution was taken as the fully relaxed or ground state energy computed using DFT. The phonon density of the states was calculated using both DFT and trained ML models to assemble and diagonalize the dynamical matrix from a numerical approximation of the PES Hessian using the built-in ASE `Phonons` module. For the MD method, the phonon density of states was extracted directly from the simulation via the Fourier transform of the velocity autocorrelation function using the `pwtools` python package. In both the FD and MD method, the internal and vibrational energies were calculated using the ASE `CrystalThermo` package. Note that the ASE module automatically normalizes the phonon density of states to match the expected vibrational degrees of freedom in the system. The density of states extracted from the MD simulations was manually normalized.

1.3 Convergence of Formation Energy

Table S1 contains the convergence information for the extended ML-only runs. These values represent the deviation of the formation energy of each alloy from the ML MD value computed after 20 ps.

Table S1: Difference between reference MD ML formation energies for the 20 ps run and MD ML formation energies computed running the simulation in predict only mode for an additional 200 ps.

Formation Energy Convergence	9.777	69.842	10.689	12.101	3.624	meV

1.4 Efficiency Data

Table S2 contains a summary of efficiency data for the SOAP and GMP models during on-the-fly training on the alloys. In every case, the efficiency of the on-the-fly procedure favors the GMP models. The GMP models require anywhere from 2-3x fewer DFT calls, 4-5x fewer training atoms, and less overall walltime. The simulation time here is dominated by DFT calls. The amount of walltime spent in ML operations is occasionally larger for GMP, but this inverts as the number of elements in the systems increases due to an increase in the walltime for SOAP models and no change in the GMP walltimes.

Table S3 contains the efficiency data for the single element systems. These results differ from the alloy systems in a couple of ways. First, the number of DFT calls is similar during the on-the-fly simulation for GMP and SOAP models. Second, the wall time spent on MLFF operations heavily favors the SOAP based models in all but one case. Interestingly, the number of columns in the GMP single element models is on par with the the number of columns in the alloy models which suggests that the GMP descriptor may be more information dense than SOAP. The SOAP models, on the other hand, contain nearly 2x fewer columns for the single element systems than the alloys. The total wall times for the GMP models are comparable to the alloy systems, whereas the wall times for the SOAP runs are

Table S2: Selected metrics for assessing model efficiency for GMP and SOAP simulations on alloys. The timings are approximate and correspond to wall times. CPU hours may be computed by multiplying the wall times by 240. The "Columns" metric refers to the number of columns in the design matrix. The number of rows corresponds direcly to the number of KS steps.

Material System	Method	No. of KS Steps Performed	WTime DFT (h)	WTime MLFF (h)	Total WTime (h)	Columns
PtAg	GMP	63	2.31	0.99	3.30	47
	SOAP	124	4.67	0.41	5.08	203
AuPtAg	GMP	55	1.78	1.00	2.78	39
	SOAP	147	5.13	0.82	5.95	209
IrPtAgAu	GMP	70	3.02	1.06	4.08	55
	SOAP	145	6.31	1.07	7.38	227
PdPtAgAuIr	GMP	49	2.11	1.01	3.12	49
	SOAP	153	6.55	2.58	9.13	230
RhPtAgAuIrPd	GMP	52	2.24	0.99	3.23	52
	SOAP	170	8.45	2.36	10.81	276

greatly reduced.

Table S3: Selected metrics for assessing model efficiency for GMP and SOAP simulations on single element bulk structures. The metrics are the same as in Table S2

Material System	Method	No. of KS Steps Performed	WTime DFT (h)	WTime MLFF (h)	Total WTime (h)	Columns
Al	GMP	33	0.25	0.74	0.99	33
	SOAP	52	0.48	0.13	0.61	129
Ag	GMP	60	1.80	0.72	2.52	31
	SOAP	59	1.64	0.09	1.73	81
Au	GMP	89	2.90	1.07	3.97	29
	SOAP	104	3.40	0.08	3.48	81
Ir	GMP	50	1.81	1.35	3.16	29
	SOAP	34	1.07	0.07	1.14	62
Pd	GMP	62	2.46	0.86	3.32	36
	SOAP	53	1.98	0.09	2.07	87
Pt	GMP	64	2.35	1.24	3.59	28
	SOAP	46	1.59	0.07	1.66	64
Rh	GMP	43	1.75	0.88	2.63	34
	SOAP	40	1.45	0.09	1.54	82

1.5 Tabulated Values of Energies

Here, we include tabulated values of internal energies and entropies (Table S4) and formation energies (Table S5) corresponding to the figures of the main text for reference, as well as tabulated values of the contributions due to configurational entropy (S6.

Table S4: Differences in internal energy and entropy at 500 K for the formation of the alloy from its constituent elements. Includes the configurational entropy of the alloy as an ideal solid solution

Number of Elements	2	3	4	5	6	eV
FD ML	-2.968360	-3.641422	-4.021960	-4.119515	-4.621012	
FD DFT	-2.797997	-3.043473	-3.748951	-4.239140	-4.393300	
FD Uncertainty Bound	-2.279862	-1.998956	-2.761698	-3.508418	-3.619274	
MD ML	-1.034114	-1.636513	-2.174891	-2.526585	-2.577619	
MD DFT	-0.966875	-1.501839	-2.060034	-2.238274	-2.406698	

Table S5: Formation Energies. Includes electronic energy as well. Equivalent to a Helmholtz free energy of formation.

Number of Elements	2	3	4	5	6	eV
FD ML	-2.045798	-3.154633	1.950198	-0.214359	-0.562661	
FD DFT	-1.875435	-2.556684	2.223207	-0.333984	-0.334949	
FD Uncertainty Bound	-1.952282	-2.580561	2.174884	-0.450343	-0.371092	
MD ML	-0.111552	-1.149723	3.797267	1.378571	1.480732	
MD DFT	-0.044313	-1.015050	3.912124	1.666882	1.651653	

Table S6: Configurational entropy corresponding to an ideal solid solution for each of the alloys considered here. The numbers were computed by assuming that 1 mol of alloy corresponds to N_{Av} atoms in the ratio equivalent to that represented in our 32 atom supercells.

Number of Elements	2	3	4	5	6	eV
Config S	0.95569	1.51337	1.91138	2.21504	2.46514	eV

1.6 Pearson Correlation Coefficients for UQ

In this work, we utilized the maximum diagonal component of the Bayesian covariance matrix as the uncertainty quantification metric. Figure S1 presents the Pearson's correlation coefficient between the maximum Bayesian error estimate for the forces and the actual RMS force errors for all on-the-fly simulations. It is clear that there is great disparity in the quality of the Bayesian estimate as a probe for the actual error in the system. Several features are of special interest. First, there do not appear to be any clear trends for the quality of the estimate. There is variation both within the single-element systems and as new species are

introduced via the alloys. Second, the absolute values of the correlations coefficients range from nearly 0 to 1 for SOAP, whereas the range for GMP is -0.5 to 0.7. It is rare that the correlation exceeds 0.6 regardless of the model type used.

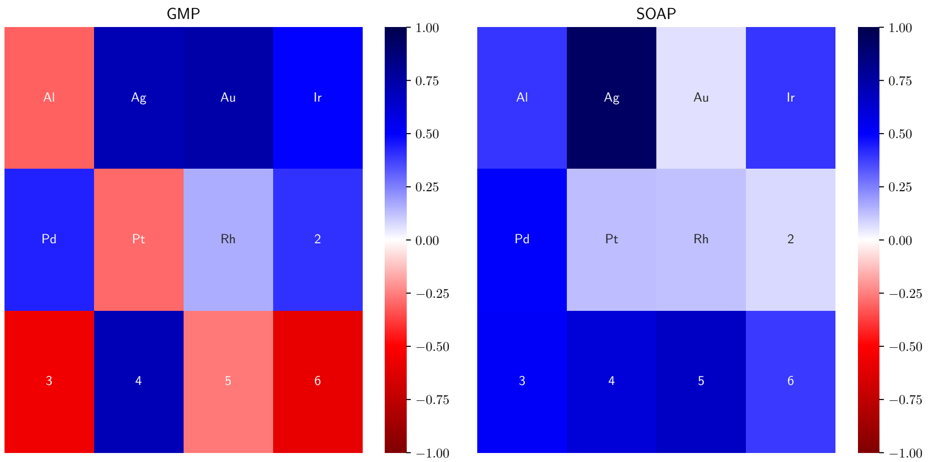

Figure S1: Heatmap of correlation coefficients for the maximum predicted error and the actual RMS error in the system.
